# Representation learning for clinical time series prediction tasks in electronic health records

**DOI:** 10.1186/s12911-019-0985-7

**Published:** 2019-12-17

**Authors:** Tong Ruan, Liqi Lei, Yangming Zhou, Jie Zhai, Le Zhang, Ping He, Ju Gao

**Affiliations:** 1 School of Information Science and Engineering, East China University of Science and Technology, 130 Meilong Road, Shanghai, 200237 China; 2 Shanghai Hospital Development Center, 2 Kangding Road, Shanghai, 200000 China; 3 Shuguang Hospital Affiliated to Shanghai University of Traditional Chinese Medicine, 528 Zhangheng Road, Shanghai, 201203 China

**Keywords:** Electronic health records, Mortality prediction, Representation learning, Recurrent neural network

## Abstract

**Background:**

Electronic health records (EHRs) provide possibilities to improve patient care and facilitate clinical research. However, there are many challenges faced by the applications of EHRs, such as temporality, high dimensionality, sparseness, noise, random error and systematic bias. In particular, temporal information is difficult to effectively use by traditional machine learning methods while the sequential information of EHRs is very useful.

**Method:**

In this paper, we propose a general-purpose patient representation learning approach to summarize sequential EHRs. Specifically, a recurrent neural network based denoising autoencoder (RNN-DAE) is employed to encode inhospital records of each patient into a low dimensional dense vector.

**Results:**

Based on EHR data collected from Shuguang Hospital affiliated to Shanghai University of Traditional Chinese Medicine, we experimentally evaluate our proposed RNN-DAE method on both mortality prediction task and comorbidity prediction task. Extensive experimental results show that our proposed RNN-DAE method outperforms existing methods. In addition, we apply the “Deep Feature” represented by our proposed RNN-DAE method to track similar patients with t-SNE, which also achieves some interesting observations.

**Conclusion:**

We propose an effective unsupervised RNN-DAE method to summarize patient sequential information in EHR data. Our proposed RNN-DAE method is useful on both mortality prediction task and comorbidity prediction task.

## Background

The past decade has witnessed an explosion in the amount of digital information recorded in electronic health records (EHRs). The EHR data is an essential resource for clinical researchers to design quantitative models, and it is crucial to understand the information contained in EHRs. In this case, machine learning models have been widely-used to analyze data with patient’s EHRs, especially for predicting health status and helping diagnose diseases, such as disease risk prediction [1], mortality prediction [2] and similarity analysis [3]. However, it is a great challenge to directly deal with raw EHR data due to its temporality, high dimensionality, noise, systematic bias, sparseness and random error [4]. Take temporality as an example, the information about the impending patient disease status is closely related to the sequence of medical events. Moreover, the same clinical phenotype may have many descriptions in EHRs [5]. Therefore, the success of predictive models relies heavily on the representation of data. In other words, extracting useful features from patient EHRs is one key aspect leading to the success of prediction models.

Representation learning methods have been used extensively within and outside the clinical domain to learn the semantics of words, phrases, and documents. For instance, Mikolov et al. [6] applied neural language models to learn a distributed representation for each word, called a word embedding. They further proposed an unsupervised algorithm [7] to learn fixed-length feature representations from variable-length pieces of texts, such as sentences, paragraphs, and documents. Peters et al. [8] used a bidirectional long short-term memory network trained on a specific task to derive word embeddings. They came up with the contextualized embedding (i.e., each word has multiple embeddings depending on the context it is used in) through grouping together the hidden states of the their model. Devlin et al. [9] proposed a language representation model called bidirectional encoder representations from transformers to generate word embeddings. Those representations perform effectively results on multiple natural language processing tasks, such as question answering and language inference. Traditional representation methods such as one-hot encoding and multi-hot encoding treat every dimension independently. Compared to the vectors generated by these methods, those derived by representation learning models are low-dimensional and dense, and they capture the semantics in context.

In the clinical domain, considerable efforts also have been made to convert medical information in EHRs to vectors. For example, Choi et al. [10] learned word embeddings of medical concepts. Nguyen et al. [11] extracted features from medical records with a convolutional neural network model. Zhou et al. [12] applied stacked denoising autoencoders [13] to learn deep representations for predictive diagnoses. These works are all based on deep learning methods. In some degree, deep learning methods can overcome the difficulties in representation learning caused by the complexity of EHRs. However, deep learning models of these works are trained to deal with a specific task rather than a general task. We have to re-learn or re-tune a new representation when giving a new predictive task.

Learning a patient representation from for general purpose is necessary to make it available for various medical prediction tasks. The main challenge is to encode the sequential information of EHRs into a vector. Considering the temporality of EHRs, each patient typically has multiple inpatient records. Since previous medical events may have an impact on future medical events, these continuous medical records are critical for clinical diagnosis and treatment.

In this paper, we propose an effective patient representation learning method for time-series prediction tasks based on real-world EHR data, which greatly improves and extends our previous work [14]. We develop a recurrent neural network based denoising autoencoder (RNN-DAE) to summarize inpatient records of each patient into a dense vector. In detail, a sub-repository for heart failure disease is first constructed from the clinical data repository of the Shuguang Hospital. After that, we represent clinical event information of a patient with a tensor, i.e., a series of multi-hot vectors. Finally, we generate patient representation vector by using our RNN-DAE model. With the help of our RNN-DAE model, time-series information in EHR data is well integrated in our patient representation. The main contributions of this paper are summarized as follows:

- We propose an effective patient representation learning method for the time-series prediction tasks in EHR data. Our proposed patient representation learning method uses recurrent neural network based denoising autoencoder (RNN-DAE) to encode time-series information. Unlike existing patient representation learning methods, our proposed RNN-DAE method considers the time series information in patient presentation.
- Based on the heart failure EHR data collected from the Shuguang Hospital, we experimentally evaluate our proposed RNN-DAE method on two clinical time series prediction tasks. Computational studies show that our proposed RNN-DAE method is highly competitive compared to existing methods, achieving an AUC of 78.31*%* in mortality prediction task and the best result in comorbidity prediction task. In addition, we apply the “Deep Feature" represented by our proposed RNN-DAE method to track similar patients with t-SNE, which also achieves some interesting results.

## Related work

In this section, we first briefly introduce state-of-the-art models for the mortality prediction and disease risk prediction task of heart failure. then, we report the progress of the representation learning methods in the medical field.

### Mortality prediction and disease risk prediction for heart failure

Mortality prediction and disease risk prediction tasks are very two essential health applications. It has been found that many factors are able to increase mortality for heart failure, such as demographic factors (e.g., gender), clinical factors (e.g., renal dysfunction), comorbidities (e.g., diabetes), cardiac imaging markers (e.g., cardio-thoracic ratio and ejection fraction) and serum biomarkers (e.g., brain natriuretic peptide and C-reactive protein). In recent years, a lot of studies have shown that machine learning methods play an important role in medical research, including support vector machine, Bayesian network, decision tree, nearest neighbors method, and ensemble learning method [15]. For instance, Lee et al. [16] proposed a mortality prediction model with a patient similarity metric. Three types of classification models were used in their work, such as logistic regression, simple statistics and decision tree. Panahiazar et al. [17] designed a risk prediction model by using support vector machine, logistic regression, random forest, adaboost and decision tree. Furthermore, some researchers [15, 18] experimentally compared and analyzed multiple mortality prediction models. The results of these works varies because their data and experiment settings are totally different, but they did actually demonstrate that machine learning methods have limitations in some degree.

Recently, deep learning methods play an important role in medical research. For example, Choi et al. [19] and Lipton et al. [20] integrated time-series information into medical applications by recurrent neural network. Nevertheless, their model focus on event-level time-series information (e.g., a series of blood pressure tests). Besides, their model is not universal and can only handle specific tasks. Cheng et al. [4] applied deep learning model to extract phenotypes from EHR data. Although the representations of phenotypes could be used in some further applications, the convolutional neural network they developed in this work might ignore the sequentiality of events. Compared with traditional machine learning models, deep learning models require less human efforts on feature engineering, but their results are more difficult to interpret.

### Representation learning in medical field

Since effective feature representation is a basic step before further applications, a large amount of studies are devoted to exploring representation learning methods in the medical field.

Inspired by the work of word embedding in natural language processing, many studies focus on representing medical concepts in recent years. For example, Minarro-Giménez et al. [21] developed skip-gram to get the representations of medical terms. Their medical texts are collected from Wikipedia, PubMed, Medscape and Merck Manuals. Choi et al. [22] learned low-dimensional vector representations of medical codes in longitudinal EHRs with skip-gram-based model. Medical codes include disease, medication and procedure codes. In their studies, patient representation with one record is generated by aggregating all the vectors of medical codes. Another study [10] proposed an approach named “Med2Vec" to learn the representations of medical codes in code level and visit level. Cui et al. [23] proposed a supervised model guided by specific prediction tasks to facilitate representations of medical codes, and it is effective to work with small EHR datasets. Deepika and Geetha [24] used a semi-supervised learning framework which contains representation learning of drugs to predict the drug interactions. However, these studies are all concept level, which means that the representations are learned to represent medical codes rather than patient representations.

Meanwhile, patient representations are widely used in several applications to assist clinical staff. Considerable efforts were made to learn dense vector representations at the patient level. For example, Zhou et al. [12] developed an unsupervised feature selection scheme relied on stacked denoising autoencoders (SDAs). However, their model aims to summarize time-series features in an inpatient record, rather than the temporality between multiple inpatient records. Miotto et al. [25] adopted SDAs to generate patient representations. Furthermore, Sushil et al. [26] derived task-independent patient representations directly from clinical notes by applying SDAs and a paragraph vector model. The above two methods only consider the frequency of medical events. The main difference between our works and theirs is that they ignore the temporality of EHRs. In addition, Zhang et al. [27] applied Bi-LSTM network to derive the patient vectors based on specific prediction. Although they take time series into consideration, this method is task-driven and supervised.

## Methods

**Fig. 1:**
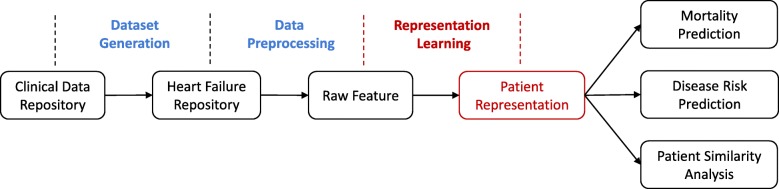
An overview of the proposed representation learning approach to generate patient vectors and further applications

The overview of our proposed patient representation learning framework and its potential applications are shown in Fig. 1. Specifically, a sub-repository focusing on heart failure is built from clinical data repository (CDR) firstly. EHR data stored in the sub-repository is then normalized and processed to tensors. Afterwards, we derive the patient representations (called “Deep Features”) by using our proposed RNN-DAE method. Finally, the obtained “Deep Features” applied for some time series prediction tasks, such as mortality prediction and comorbidity prediction. We use “Deep Feature” to conduct patient similarity analysis as well.

### Dataset generation: heart failure selection

The EHR data used in this paper is collected from the Shuguang Hospital which is the first class general hospitals in Shanghai. The CDR of the Shuguang Hospital between January 2005 and April 2016 contains approximately 350,000 hospital records.

In this paper, a sub-repository focusing on heart failure is constructed from the above CDR. We select patients who satisfy the following criteria: One patient has at least two hospital records, and the ICD-10 code associated with heart failure exists in the diagnosis or medical order of these two hospital records. Specially, clinical experts define a list of ICD-10 codes related to heart failure, including 63 codes.

Our dataset consists of 4682 patients with 10,898 inpatient records, where 568 patients (about 12.1*%*) died in the hospital and the remaining patients are difficult to track. To enrich our dataset, we split the patients’ hospital records and obtain 10,898 samples. For instance, if a patient has three inpatient records, we then construct three samples by respectively selecting only the first record, both the first and second records, and all three records.

### Data preprocessing

For each patient in the sub-repository, auxiliary information, general demographic details (i.e., age and gender), and clinical events are retained. Auxiliary information contains EMPI (i.e., patient unique identifier), hospital ID (i.e., inpatient record unique identifier), admission time and death time. We use auxiliary information to organize and preprocessing EHR data. General demographic details (i.e., age and gender) only needs two dimensions to describe, and the value of age should be normalized without breaking sparsity first. Besides, clinical events include diagnoses, medications and lab tests. To convert clinical events to computable sequences, the normalization process for different clinical events varies by their types. In particular, we convert clinical event information of one record to a multi-hot vector. Finally, a multi-hot vector with 1309 dimensions is obtained according to the following principles:

- Diagnoses: The patient records of heart failure repository include 1232 ICD-10 codes in total. As a result, we represent the ICD-10 codes with 1232 dimensions.
- Medications: According to the universality of medication for heart failure in China, 61 kinds of medications are chosen by clinical specialists manually. Clinical specialists classified these medications into 11 groups, such as ACE-I, ARA, and ARB. Similarly, we represent the medications with 11 dimensions.
- Lab Tests: Clinical experts choose 22 laboratory tests related to heart failure in this research. According to the reference value of each lab test, a flag including high, low and normal is used to denote the results. Therefore, three dimensions are required to convert the result of one lab test into binary feature. Eventually, we represent the lab tests with 66 dimensions.

Specially, raw feature includes clinical events and demographic details, and one record of raw feature is described with 1311 dimensions in total.

### Patient representation learning

**Fig. 2:**
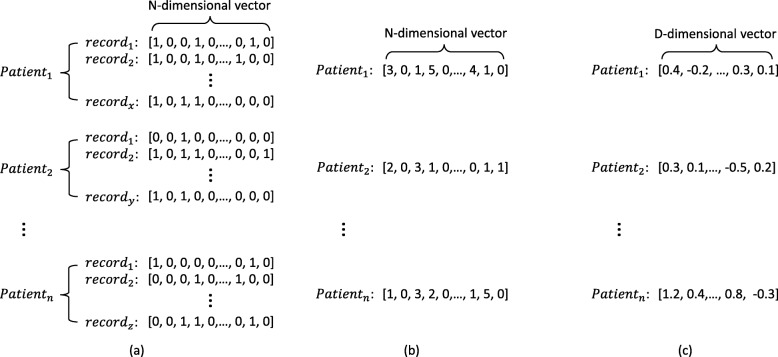
Three different forms of the representation of patients. Here, patient may have various inpatient times (e.g., *x,y*,*z*). The tensor representation of each patient consists of multiple multi-hot vectors of *N*-dimensions (i.e., *N*=1309). The statistic-based representation is derived by operating summary statistics, and it gets a vector with *N*-dimensions. Typically, distributed representation is a better representation with *D*-dimensions (i.e., *D*=300), where *D* is much lower than *N*. **a** Tensor representation of patients. **b** Statistic-based representation of patients. **c** Distributed representation of patients

Figure 2 describes a straightforward motivation for using distributed representation for patients. The size of tensor representations is variable because different patients may have various inpatient times(i.e., *x,y* or *z* times). As shown in Fig. 2a, it is challenging to use the tensors with variable length as the input of prediction models. To solve this issue, the representation method in Fig. 2b performs statistics for all the inpatient records of each patient, such as summarize, average, and maximize. For example, the value on each dimension of the patient vector is the summary of the corresponding medical event in all inpatient records. Therefore, the dimensions of patient vector is equal to the number of distinct medical events appeared in the raw data. However, these kind of representation is still high dimensional and sparse. Moreover, they do not take the time series information in EHRs into consideration. A better way to represent patients is shown in Fig. 2c. By using RNN-DAE model, we will use distributed representation to better represent patients as multi-dimensional real-valued vectors that will capture the time series information between records.

Given a sequence of inpatient records *X*=(***x***_1_,***x***_2_,⋯,***x***_*n*_), where ***x***_*t*_(*t*=1,⋯,*n*) is a multi-dimensional multi-hot vector which represents an inpatient clinical event record at time step *t*, our goal is to summarize a feature vector representation ***c*** from these sequence of clinical events. Finally, ***c*** will be concatenated with demographic details to get our “Deep Feature”.

RNN is widely used to cope with time-series prediction problems [28, 29]. RNN can remember historical information because the value of current hidden layer depends on the input of current layer and the output of previous layer. Based on the standard RNN, Hochreiter et al. [30] proposed long short-term memory (LSTM) model to cope with gradient exploding and vanishing problems [31, 32]. To simplify the structure of LSTM, one of the most popular variants is gated recurrent unit (GRU) model [33] is developed. The GRU model keeps both advantages of RNN and LSTM, that is, supporting longer sequences but consuming less training time [34]. Therefore, we replace the standard RNN unit with GRU in our research.

**Fig. 3:**
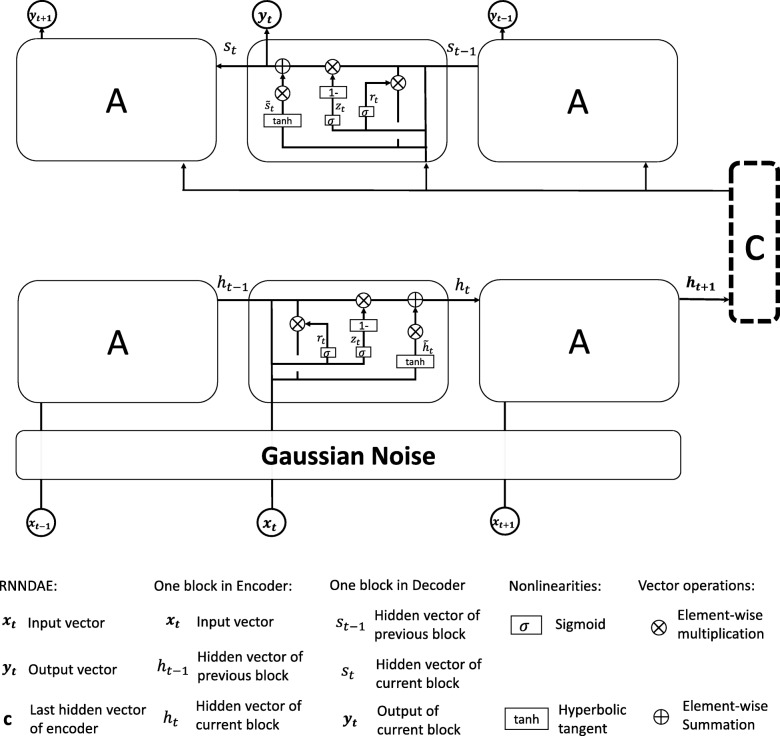
The architecture of our proposed RNNDAE model. Multi-hot vectors (***x***_*t*_) with time series are added by a Gaussian noise and then encoded by a ***GRU***_*encoder*_ model into the patient vector (***c***). Given the patient vector, another ***GRU***_*decoder*_ model is used to decode in order to make the input (***x***_*t*_) and the output (***y***_*t*_) are consistent as much as possible

We develop a recurrent neural network based denoising autoencoder (RNN-DAE) model in this paper, which combines the ideas of SDAs [13] and sequence autoencoders [35]. In detail, our model trains a ***GRU***_*encoder*_ to convert input features to a vector, and then a ***GRU***_*decoder*_ is developed to predict input features sequentially. Specially, the decoder reconstructs the initial inputs from a noisy version of the input features. Figure 3 illustrates the architecture of our RNN-DAE model.

In order to avoid over-fitting when train our model, input vectors ***X*** are first mapped through a stochastic mapping $\boldsymbol {\tilde {X}} \thicksim \boldsymbol {q_{D}(\tilde {X}|X)}$. Specially, we adopt Gaussian noise as the stochastic mapping to get $\boldsymbol {\tilde {X}}$. Gaussian noise is a series of random numbers with a Gaussian distribution. The ***GRU***_*encoder*_ reads the $\boldsymbol {\tilde {X}}$ and turn it into a vector ***c***, where ***c*** is actually the last hidden state of ***GRU***_*encoder*_ which summarize the whole input sequence. The ***GRU***_*encoder*_ predicts the next state ***h***_*t*_ at time step *t* given the input ***x***_*t*_ and the previous hidden state ***h***_*t*−1_ as follows:

1 $$ \boldsymbol{z}_{t} = \boldsymbol{\delta}(\boldsymbol{W}_{z}\cdot[\boldsymbol{h}_{t-1},\boldsymbol{x}_{t}])  $$

2 $$ \boldsymbol{r}_{t} = \boldsymbol{\delta}(\boldsymbol{W}_{r}\cdot[\boldsymbol{h}_{t-1},\boldsymbol{x}_{t}])  $$

3 $$ \boldsymbol{\tilde{h}}_{t} = \boldsymbol{tanh}(\boldsymbol{W}\cdot[\boldsymbol{r}_{t}\ast\boldsymbol{h}_{t-1},\boldsymbol{x}_{t}])  $$

4 $$ \boldsymbol{h}_{t} = (1-\boldsymbol{z}_{t})*\boldsymbol{h}_{t-1}+\boldsymbol{z}_{t}*\boldsymbol{\tilde{h}}_{t}  $$

where ***r***_*t*_ is the reset gate, ***z***_*t*_ is the update gate, ***δ(·)*** indicates a sigmoid activation function, and ***tanh(·)*** represents a tangent activation function. The reset gate reads the values of ***h***_*t*−1_ and ***x***_*t*_ and outputs the values (between 0 to 1) to the state ***h***_*t*−1_ of each cell through the Eq. (2). The update gate updates the hidden state to the new state ***h***_*t*_.

After encoding, ***GRU***_*decode*_ is used to predict the next state ***y***_*t*_ at time step *t* based on the global patient vector ***c*** and the previous hidden state ***s***_*t*−1_ as follows:

5 $$ \boldsymbol{z}_{t} = \boldsymbol{\delta}(\boldsymbol{W}_{z}\cdot[\boldsymbol{s}_{t-1},\boldsymbol{c}])  $$

6 $$ \boldsymbol{r}_{t} = \boldsymbol{\delta}(\boldsymbol{W}_{r}\cdot[\boldsymbol{s}_{t-1},\boldsymbol{c}])  $$

7 $$ \boldsymbol{\tilde{s}}_{t} = \boldsymbol{tanh}(\boldsymbol{W}\cdot[\boldsymbol{r}_{t}*\boldsymbol{s}_{t-1},\boldsymbol{c}])  $$

8 $$ \boldsymbol{s}_{t} = (1-\boldsymbol{z}_{t})*\boldsymbol{s}_{t-1}+\boldsymbol{z}_{t}*\boldsymbol{\tilde{s}}_{t}  $$

9 $$ \boldsymbol{y}_{t} = \boldsymbol{s}_{t}  $$

where ***s***_*t*_ is the hidden state of the decoder at time *t*.

Reconstruction error *L*(*x,y*) is defined as the loss function, and the model optimize the parameters by minimizing reconstruction error. We utilize cross-entropy function to calculate the reconstruction error as follows:

10 $$ {}L(X, Y)\!\! =\! -\sum_{i=1}^{n} \sum_{j=1}^{d} \left[x_{i}^{(j)}\log y_{i}^{(j)} + \left(1-x_{i}^{(j)}\right)\log\left(1-y_{i}^{(j)}\right)\right]  $$

where $x_{i}^{(j)}$ is the *j*-th element of ***x***_*i*_ and $y_{i}^{(j)}$ is the *j*-th element of ***y***_*i*_. *d* is the dimension of ***x***_*i*_ and ***y***_*i*_.

The Gaussian noise is set with a mean of 0 and a variance of 0.1. The output dimensions of ***GRU***_*encoder*_ and ***GRU***_*decoder*_ are all 300, therefore, ***c*** is a 300-dimensional vector. When training the network, the loss is minimized by gradient-based optimization with mini-batch of size 100.

Finally, each patient vector consists of 302 dimensions and is renamed as “Deep Feature". Among them, 2 dimensions are demographic details (i.e., age and gender), and the other 300 dimensions are the output of our representation model (i.e., RNN-DAE). We do not input the demographic details into our models because they are of great significant effect on clinical tasks. The vector ***c*** is derived by encoding clinical events only.

## Results

We compared our RNN-DAE model with other well-known feature learning methods on mortality prediction and comorbidity prediction tasks. Traditional methods such as *k*-means clustering(i.e., *k*-means),principal component analysis (PCA), and Gaussian mixture model (GMM)[36] performed only one transformation to the original data, while deep learning method (i.e., SDAs) needs to perform three transformations. The details of traditional models to perform representation learning are as follows.

- PCA uses an orthogonal transformation to convert a set of observations of possibly correlated variables (entities each of which takes on various numerical values) into a set of values of linearly uncorrelated variables called principal components. This transformation is defined in such a way that the first principal component has the largest possible variance (that is, accounts for as much of the variability in the data as possible), and each succeeding component in turn has the highest variance possible under the constraint that it is orthogonal to the preceding components. The resulting vectors are an uncorrelated orthogonal basis set, and the dimensions of them are less than or equal to that of original data. Here, we set the PCA with 512 principal components.
- *k*-means clustering aims to partition unlabeled data into k clusters in which each observation belongs to the cluster with the nearest mean. In the feature learning, the centroids of the cluster are used to produce features. Specially, we used the vector of centroid of the cluster to represent the data within this cluster in our experiments and we set *k*-means with 16 clusters.
- GMM is a probabilistic model that assumes all the data points are generated from a mixture of a finite number of Gaussian distributions with unknown parameters. One can think of mixture models as generalizing *k*-means clustering to incorporate information about the covariance structure of the data as well as the centers of the latent Gaussian. Each component (i.e., Gaussian distribution) of GMM is a clustering center and has its own diagonal covariance matrix. In the GMM model, the number of components needs to be artificially defined, just like the clusters number in the *k*-means. Specially, we used the vector of the covariance of each component to represent the data within this cluster in our experiments and we set GMM with 512 components.

In this section, we devote to experimentally investigate the effectiveness of our proposed RNN-DAE method. Besides RNN-DAE, we also evaluate a variant of RNN-DAE method. That is a RNN based autoencoder model without Gaussian noise (RNN-AE). RNN-AE model is an alternative of RNN-DAE by removing Gaussian noise. In following experiments, we applied our proposed methods to mortality prediction task, comorbidity prediction task, and patient similarity analysis. Experimental results are recorded in terms of Accuracy, F_1_-score and the area under the curve (AUC), they are widely-used performance measures [37, 38].

### Mortality prediction

Our proposed model is compared with four state-of-the-art methods. Three of them are based on traditional machine learning model including GMM, PCA and *k*-means, and the remaining one is based on deep learning model called “SDAs” [12]. Furthermore, we add an ablation experiment to investigate the effect of the proposed denoising part. In other words, we also develop a RNN-AE model without Gaussian noise. According to patient vectors derived from above representation learning models, downstream classifier is used to predict mortality. The comparison of different downstream classifiers are performed in the “Discussion” section.

Due to traditional machine learning models can not deal with sequential data directly, observation windows are required to extract features. In order to investigate impact of window sizes, we conduct the experiments to compare the performance of representation learning models under various window sizes. Specially, the comparison is made on mortality prediction task. According to the studies [19, 25], the window sizes are set with 30, 60, 90 and 180 days. Table 1 shows experimental results. The first column includes a series of represent learning methods, where “Hand" indicates that the raw features of each patient are only averaged. Since our proposed models RNN-DAE and RNN-AE do not rely on window size, they achieve 0.783 and 0.755 respectively on AUCs in all cases. As the size of the window grows, the performance of representation learning models based on traditional machine learning methods will increase as well. The reason is that the larger the window size, more records it covers and more useful information it provides. Consequently, we set the window size with 180 days in later experiments. The performance of comparison methods grows stably on AUC, but our RNN-DAE model is at least 15.5*%* better than traditional machine learning models and 2.8*%* better than the deep learning method “SDAs”. Comparative results of different representation learning models for mortality prediction task are summarized in Table 2. For the mortality prediction task, we set the threshold value as 0.8 for classification. The result shows that our RNN-DAE model with Gaussian noise outperforms other models remarkably, achieving 0.783 on AUC, 0.779 on accuracy and 0.449 on F_1_-score.

**Table 1:** Comparative results of methods with different window sizes

	30 Days	60 Days	90 Days	180 Days
RNN-DAE	0.783	0.783	0.783	0.783
RNN-AE	0.755	0.755	0.755	0.755
SDAs	0.488	0.738	0.741	0.755
Hand	0.525	0.584	0.586	0.608
PCA	0.504	0.555	0.555	0.602
GMM	0.536	0.595	0.594	0.607
*k*-means	0.569	0.568	0.568	0.628

The performance is measured by AUC (i.e., the area under the ROC curve).

**Table 2:** Comparative results of different representation learning methods for mortality prediction

	AUC	Threshold =0.8	
		Accuracy	F_1_-score
RNN-DAE	0.783	0.779	0.449
RNN-AE	0.755	0.760	0.444
SDAs	0.755	0.738	0.439
Hand	0.608	0.693	0.420
PCA	0.602	0.715	0.427
GMM	0.607	0.693	0.420
*k*-means	0.628	0.722	0.430

### Comorbidity prediction

Comorbidity prediction task is a typical disease risk prediction task. In this experiment, we consider ten comorbidities related to heart failure, and further validate the effectiveness of our RNN-DAE method on comorbidity prediction task. The statistical results of comorbidities are shown in Table 3. Several comorbidities are so rare in the dataset, and need to undersample when training classifiers. For example, only 80 patients with valvular heart disease occur. The column “Count" represent the number of occurrences of each comorbidity and the column “Percent" indicates the percentage of each comorbidity in our dataset. For these comorbidities with percentage is less than 30%, we apply NearMIss undersampling algorithm before classification [39]. At the last column “Sample?", we also indicate the use of sampling algorithm or not.

**Table 3:** Statistics of 10 selected comorbidities in heart failure

Comorbidity	Count	Percent	Sample?
Hypertension disease	7,097	0.694	n
Diabetes mellitus	3,674	0.359	n
Coronary artery disease	5,072	0.496	n
Atrial fibrillation	3,053	0.299	y
Chronic renal disease	896	0.088	y
Valvular heart disease	80	0.008	y
Dilated cardiomyopathy	321	0.031	y
Hypertrophic cardiomyopathy	146	0.014	y
Chronic obstructive pulmonary disease	818	0.080	y
Cerebral infarction disease	2,579	0.252	y

In the experiments, we train downstream classifiers for each comorbidity prediction task respectively based on patient vectors derived from various representation learning models. The comparison of different downstream classifiers are detailed in the “Discussion” section. Table 4 illustrates the comparative results between the patient vectors learned by seven representation models with complete ranking information. The result shows that no single model achieves optimal performance across all 10 tasks. Our RNN-DAE model achieves the most competitive performance, and RNN-AE model achieves the second highest performance. What is more, RNN-DAE model achieves the highest score on 4 out of 10 comorbidity prediction tasks, and obtains the smallest average ranks 2.000 (2.500, 5.600, 5.800, 5.400, 3.250 and 3.450 are respectively obtained by the reference algorithms RNN-AE, SDAs, PCA, *k*-means, GMM and Hand). Unlike RNN-DAE model, traditional machine learning models and the unsupervised deep learning model “SDAs” are constrained by window size. To sum up, our proposed RNN-DAE model is a better choice for comorbidity prediction task because of its better performance.

**Table 4:** Comparative results of different methods for comorbidity prediction

	RNN-DAE	RNN-AE	SDAs	PCA	*k*-means	GMM	Hand
HD	0.745(2)	0.771(1)	0.736(3)	0.545(6)	0.537(7)	0.652(5)	0.654(4)
DM	0.671(1)	0.660(2)	0.422(7)	0.619(6)	0.631(3)	0.627(4.5)	0.627(4.5)
CAD	0.744(2)	0.746(1)	0.609(6)	0.601(7)	0.617(5)	0.741(3)	0.740(4)
AF	0.743(1)	0.522(6)	0.537(4)	0.535(5)	0.404(7)	0.645(2)	0.644(3)
CRD	0.700(2)	0.727(1)	0.554(6)	0.373(7)	0.566(5)	0.699(3)	0.698(4)
VHD	0.586(5)	0.842(3)	0.601(4)	0.258(7)	0.500(6)	0.882(2)	0.902(1)
DCM	0.785(1)	0.777(2)	0.406(7)	0.416(6)	0.440(5)	0.675(3)	0.674(4)
HCM	0.718(2)	0.814(1)	0.201(7)	0.222(6)	0.396(5)	0.438(3)	0.437(4)
COPD	0.747(1)	0.547(3)	0.522(5)	0.577(2)	0.457(7)	0.522(5)	0.522(5)
CID	0.790(3)	0.739(5)	0.474(7)	0.697(6)	0.762(4)	0.872(2)	0.873(1)
Avg.rank	2.000	2.500	5.600	5.800	5.400	3.250	3.450

^⋆^Ten selected comorbidities of heart failure are hypertension disease (HD), diabetes mellitus (DM), coronary artery disease (CAD), atrial fibrillation (AF), chronic renal disease (CRD), valvular heart disease (VHD), dilated cardiomyopathy (DCM), hypertrophic cardiomyopathy (HCM), chronic obstructive pulmonary disease (COPD) and cerebral infarction disease (CID)

Furthermore, we also apply the patient vectors derived from our proposed model to predict top *k* comorbidities that a patient may suffer from. We evaluate the accuracy of top-*k* comorbidities prediction (with *k*=1,2,3). The accuracy of the downstream classifier is the average of the top-*k* accuracy of all patients. Specially, the downstream classifier assigns top *k* comorbidities to one patient by predicting the greatest *k* comorbidity scores, and the top-*k* accuracy of one patient is the correct rate in the predicted top *k* comorbidities. In this experiment, we evaluate the theoretical upper bound of the classifier for each comparison. That is, the accuracy when the classifier assigns all the correct comorbidities to each patient. However, the upper bond of top-3 comorbidities prediction is less than 1 when there is one patient with only one comorbidity in our dataset. As shown in Table 5, our RNN-DAE model performs a little worse than our original RNN-AE model in top-1, but outperforms in top-2 and top-3 prediction tasks.

**Table 5:** Prediction accuracy of top-*k* comorbidity

Top-*k* ^∘^	Upper Bound ^⋆^	Patient representation learning methods						
		RNN-DAE	RNN-AE	SDAs	PCA	*k*-means	GMM	Hand
*k*=1	0.962	0.604	0.617	0.212	0.449	0.181	0.607	0.605
*k*=2	0.878	0.534	0.514	0.195	0.384	0.208	0.503	0.503
*k*=3	0.769	0.452	0.419	0.177	0.305	0.144	0.416	0.417

^∘^“Top-*k*” represents the average accuracy of all the patients, where accuracy for one patient is the average number of correct results included in its top k predicted comorbiditie(s). The top-*k* comorbiditie(s) is/are sorted by predicted probabilities, with *k*=1,2,3.

^⋆^“Upper Bound” shows the best results achievable (i.e., all the correct comorbidities assigned to all the patients)

### Patient similarity analysis

Due to diagnosis and treatment highly relying on previous experiences, it’s important to find those patients whose physical status are similar. It helps clinicians give accurate treatments. Researchers have made a large amount of efforts [40–42] to identify patients with similar status. We make an assumption before we conduct the experiment. That is, the patients who are dead in our dataset are supposed to be similar. Based on the assumption, we try to find out patients with similar outcomes (i.e., death) using “Deep Feature” learned by our RNN-DAE model.

**Fig. 4:**
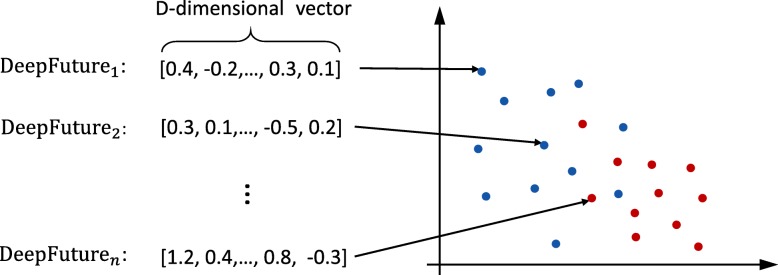
A diagram of t-SNE technique for dimensionality reduction. With the help of t-SNE, some *D*-dimensional data points are projected into 2-dimensional space. Specially, the red points indicate the patients who finally die and the blue ones represent those patients who do not die

**Fig. 5:**
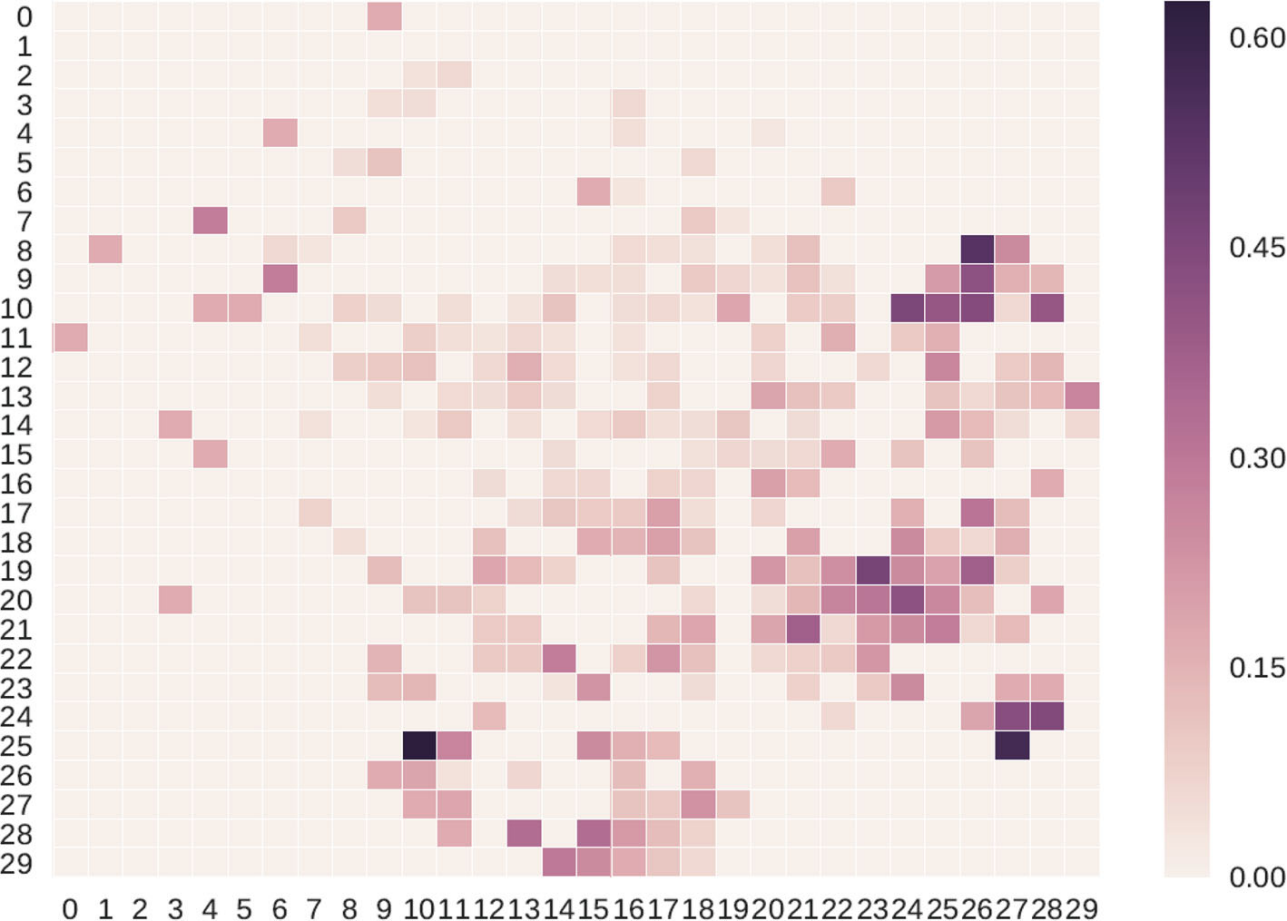
Results of patient similarity analysis based on “Deep Feature”

We use t-SNE [43] method to project “Deep Feature” of 10,898 patient records to a 2-dimensional space firstly. The t-SNE method is good at capturing much of the local structure of the high-dimensional data, while also revealing global structure. As shown in Fig. 4, the red points indicate the patients who finally die and the blue ones represent those patients who do not die. By using t-SNE, we can convert “Deep Futures” in *R*^*D*^ vector space into *R*^2^ vector space. It can capture the similarity of those “Deep Future” so that the patients who die and those not are clustered respectively. In detail, we split 2-dimensional space into 30∗30=900 blocks. For each block at location (*i,j*), the calculation of its mortality rate is performed as follows.

11 $$ H_{ij} = K_{ij}/N_{ij}  $$

12 $$ H_{ij} = K_{ij}/(N_{ij}+F)  $$

where *K*_*ij*_ indicates the number of dead records and *N*_*ij*_ represents the amount of inpatient records. When calculate the mortality rate by Eq. 11, the corresponding mortality rate will be 1.0 if a block has only one inpatient record and it is a dead one. To avoid this problem, we add *F* as a smooth factor as shown in Eq. 12, and we set 5 as an empirical value. Once we get the mortality rate of all blocks, we can construct a heatmap (see Fig. 5). The higher the mortality rate of a block, the darker the color is supposed to be. As shown in this figure, the dead records are clustered into a few blocks, and some of them have mortality rates over 60%. These interesting observations show that our “Deep Feature” is useful to calculate and visualize the similarities between patients.

## Discussion

In this section, we conduct four groups of experiments. In first three experiments, we analyze different sampling strategies, different binary classifiers, and patient representation vectors with different dimensions, respectively. Finally, we experimentally analyze the effect of different training data sizes.

### Analysis of different undersampling algorithms

**Fig. 6:**
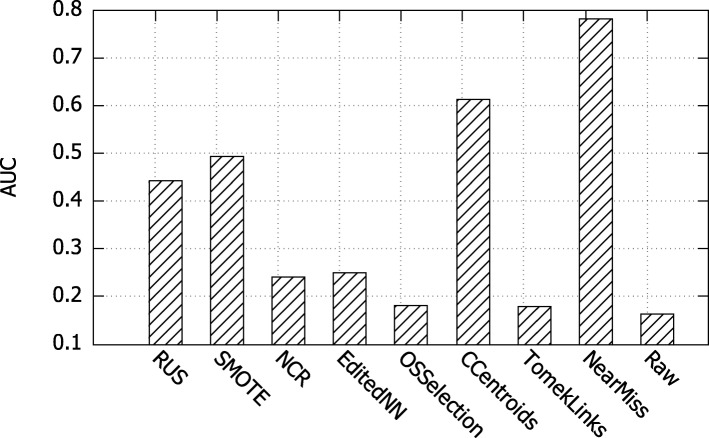
Comparative results of different sampling strategies

The death information of EHR data is usually incomplete because only patients died in hospital were recorded. Our dataset has imbalance issue because it contains 4296 patients with 583 dead ones. The same problem also exists between common diseases and rare diseases in comorbidity prediction tasks. Thus, it is necessary to undersample the dataset before the prediction tasks. Various well-known undersampling algorithms are evaluated in this experiment. Experimental results are displayed in Fig. 6, where x-axis represents different undersampling algorithms and y-axis indicates the performance in terms of AUC. Besides, “Raw" indicates that raw dataset was used without undersampling. We observe that NearMIss algorithm outperforms other undersampling strategies. As a result, we adopt NearMIss algorithm when undersampling in mortality prediction and comorbidity prediction tasks.

### Analysis of different binary classifiers

We conduct two experiments to analyze different binary classifiers. One is to determine a good classifier for downstream prediction tasks. The other is to verify the general purpose of our RNN-DAE model.

**Fig. 7:**
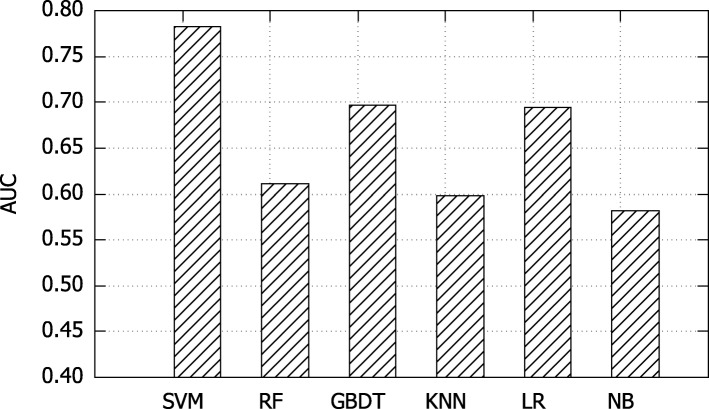
Comparative results of different binary classifiers

We compare six well-known binary classifiers based on mortality prediction task. These binary classifiers include support vector machine (SVM), random forest (RF), gradient boosting decision tree (GBDT), *k*-nearest neighbor (KNN), logistic regression (LR) and naive Bayes (NB). Figure 7 records the results, where x-axis represent various classifiers and y-axis indicates the performance in terms of AUC. As shown in this figure, SVM classifier achieves the best performance. Therefore, SVM classifier is used in both mortality prediction and comorbidity prediction tasks.

To verify that our RNN-DAE model is insensitive by the selected classifier. We experimentally compare the results of mortality prediction between various representation learning methods using different classifiers, and their results are summarized in Table 6. From this table, we observe that our proposed RNN-DAE method outperforms the traditional representation learning methods in terms of AUC, with 4 of the 6 classifiers achieving the best results. That is, our proposed RNN-DAE model is able to achieve competitive results even without the best classifier for downstream tasks.

**Table 6:** Comparative results of various representation learning methods using different classifiers for mortality prediction

	SVM	RF	GBDT	KNN	LR	NB
PCA	0.602	0.538	0.654	0.566	0.561	0.481
*k*-means	0.628	0.565	0.570	0.527	0.642	0.500
GMM	0.602	0.637	0.735	0.561	0.649	0.502
RNN-DAE	0.783	0.611	0.697	0.598	0.694	0.516
Hand	0.608	0.637	0.737	0.561	0.649	0.502

The results are measured by AUC

### Analysis of patient representation vectors with different dimensions

**Fig. 8:**
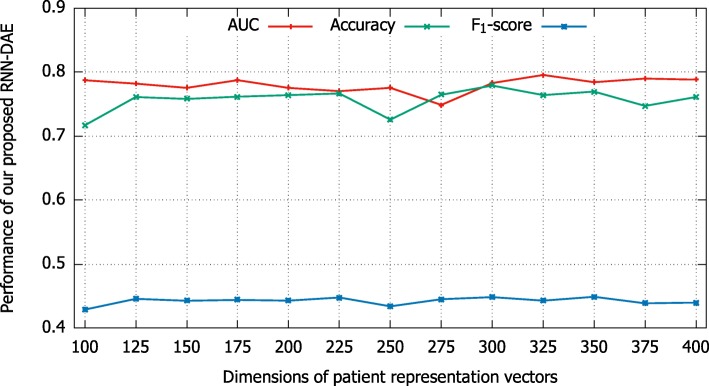
Comparative results of patient representation vectors with different dimensions

To investigate the sensitivity of our proposed model, we experimentally compare patient representation vectors with different dimensions on mortality prediction task. As shown in Fig. 8, the x-axis indicates different dimensions of patient representation vector from 100 to 400 and the y-axis denotes the performance of our proposed model in term of AUC, Accuracy and F_1_-score. We observe that the performance of our proposed model is basically stable, although it is a bit fluctuating. In other words, no matter how we vary the dimensions of our patient representation vector, the value of AUC, Accuracy, and F_1_-score can be better than 0.75, 0.71, and 0.42 respectively.

### Analysis of different training data sizes

**Fig. 9:**
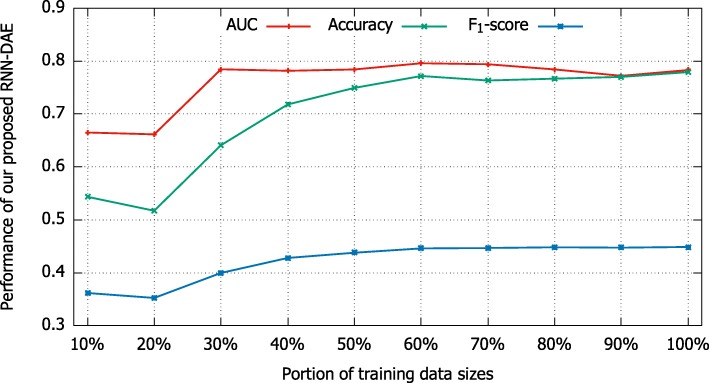
The effect of different training data sizes

To find an empirical training data size to train our proposed RNN-DAE model, we experimentally investigate the effect of different training data sizes on mortality prediction task. There are totally 10,898 samples in the training data. In the experiment, we randomly selected 10%, 20%, ⋯, 100% of 10,898 samples to train our model. Comparative results are shown in Fig. 9. From this figure, we observe that the performance of our RNN-DAE method in terms of AUC, Accuracy, and F_1_-score increases significantly when the training data increases from 10% to 30%. When the training data size continues to increase, the value of AUC comes into a steady stage, but the values of accuracy and F_1_-score continue to rise until the training data size reaches 60%. These interesting observations confirm the robustness of our proposed RNN-DAE method. That is, our RNN-DAE model is able to achieve comparable results even if only a few training data is used to train.

## Conclusions

We present an effective patient representation learning method for time-series prediction tasks in real-world EHR data. With the help of our patient representation learning method, some predictive descriptors called “Deep Features” can be derived from the EHR data. Our proposed patient representation learning method uses recurrent neural network based denoising autoencoder (RNN-DAE) to encode time-series information. Our proposed RNN-DAE method is able to capture hierarchical regularities, dependencies, and time series information in the data to create a compact, general-purpose set of patient features that can be effectively used in predictive clinical time series tasks. Based on the real-world heart failure EHR data collected from the Shuguang Hospital, we experimentally evaluate the effectiveness of our proposed RNN-DAE method on both mortality prediction task and comorbidity prediction task. In addition, we apply our proposed RNN-DAE method to conduct patient similarity analysis. Experimental results show that “Deep Features” derived by our RNN-DAE method are consistently better than those obtained by other feature learning methods based on EHR data.

In future work, we plan to investigate some possible applications of our proposed RNN-DAE method to analyze other special diseases, such as diabetes and colorectal cancer, and to solve other clinical tasks, such as personalized prescriptions and therapy recommendation. Since the patient’s inpatient records in our dataset rarely exceeds 180 days, we did not consider the window size for more than 180 days in this paper. We plan to consider window sizes over 180 days in the future.

## Data Availability

Our datasets are not publicly available due to a concern to protect individual patient confidentiality but they are available from the corresponding author on reasonable request.

## Abbreviations

AF: Atrial fibrillation
AUC: The area under the curve
Bi-LSTM: Bidirectional long short-term memory
CAD: Coronary artery disease
CDR: Clinical data repository
CID: Cerebral infarction disease
COPD: Chronic obstructive pulmonary disease
CRD: Chronic renal disease
DCM: Dilated cardiomyopathy
DM: Diabetes mellitus
EHRs: Electronic health records
GBDT: Gradient boosting decision tree
GMM: Gaussian mixture model
GRU: Gated recurrent unit
HCM: Hypertrophic cardiomyopathy
HD: Hypertension disease
ICD-10: International classification of disease version 10
*k*-means: *k*-means clustering
KNN: *k*-nearest neighbor
LR: Logistic regression
LSTM: Long short-term memory
NB: Naive Bayes
PCA: Principal component analysis
RF: Random forest
RNN-AE: RNN based autoencoder model without Gaussian noise
RNN-DAE: Recurrent neural network based denoising autoencoder
SDAs: Stacked denoising autoencoders
SVM: Support vector machine
VHD: Valvular heart disease
